# Co-occurrence of four nucleotide changes associated with an adult mitochondrial ataxia phenotype

**DOI:** 10.1186/1756-0500-7-883

**Published:** 2014-12-08

**Authors:** Ramón Zabalza, Anssi Nurminen, Laurie S Kaguni, Rafael Garesse, M Esther Gallardo, Belén Bornstein

**Affiliations:** Servicio de Neurología, Hospital Universitario de Donostia, Donostia, Spain; Institute of Biosciences and Medical Technology, University of Tampere, 33014 Tampere, Finland; Department of Biochemistry and Molecular Biology, and Center for Mitochondrial Science and Medicine, Michigan State University, East Lansing, MI 48824 USA; Departamento de Bioquímica, Facultad de Medicina, Instituto de Investigaciones Biomédicas “Alberto Sols” UAM-CSIC and Centro de Investigación Biomédica en Red (CIBERER), Madrid, Spain; Instituto de Investigación Sanitaria Hospital 12 de Octubre (i + 12), Madrid, Spain; Instituto de Investigación Hospital Puerta de Hierro Majadahonda (IDIPHIM), Madrid, Spain; Servicio de Bioquímica, Hospital Universitario Puerta de Hierro, c/Manuel de Falla 2, 28222 Majadahonda, Madrid

**Keywords:** POLG, Ataxia, Mitochondrial disorders, MIRAS

## Abstract

**Background:**

Mitochondrial DNA maintenance disorders are an important cause of hereditary ataxia syndrome, and the majority are associated with mutations in the gene encoding the catalytic subunit of the mitochondrial DNA polymerase (DNA polymerase gamma), *POLG*. Mutations resulting in the amino acid substitutions A467T and W748S are the most common genetic causes of inherited cerebellar ataxia in Europe.

**Methods:**

We report here a *POLG* mutational screening in a family with a mitochondrial ataxia phenotype. To evaluate the likely pathogenicity of each of the identified changes, a 3D structural analysis of the PolG protein was carried out, using the Alpers mutation clustering tool reported previously.

**Results:**

Three novel nucleotide changes and the p.Q1236H polymorphism have been identified in the affected members of the pedigree. Computational analysis suggests that the p.K601E mutation is likely the major contributing factor to the pathogenic phenotype.

**Conclusions:**

Computational analysis of the PolG protein suggests that the p.K601E mutation is likely the most significant contributing factor to a pathogenic phenotype. However, the co-occurrence of multiple *POLG* alleles may be necessary in the development an adult-onset mitochondrial ataxia phenotype.

## Background

Disorders that affect the maintenance of mitochondrial DNA (mtDNA) are characterized in postmitotic tissues by depletion or multiple deletions in mtDNA [1–3]. To date, mutations in twelve nuclear-encoded genes have been described in association with these clinical syndromes [4]. Mutations in one of these, the *POLG* gene encoding the catalytic subunit of the mitochondrial DNA polymerase, DNA polymerase gamma, is the most common nuclear gene causing mitochondrial disorders. In fact, more than 170 variants have been reported in the DNA polymerase gamma mutation database [5]. Clinical phenotypes associated with *POLG* mutations are diverse, ranging in severity from progressive external ophthalmoplegia [6], to mitochondrial recessive ataxia-syndrome [7–10], to Alpers–Huttenlocher syndrome [11]. We report here a familial adult-onset ataxia syndrome, associated with three novel *POLG* nucleotide variants. A computational analysis of PolGA was carried out, and suggests that one of these is most likely pathogenic.

## Methods

### Patient evaluation

**Figure 1:**
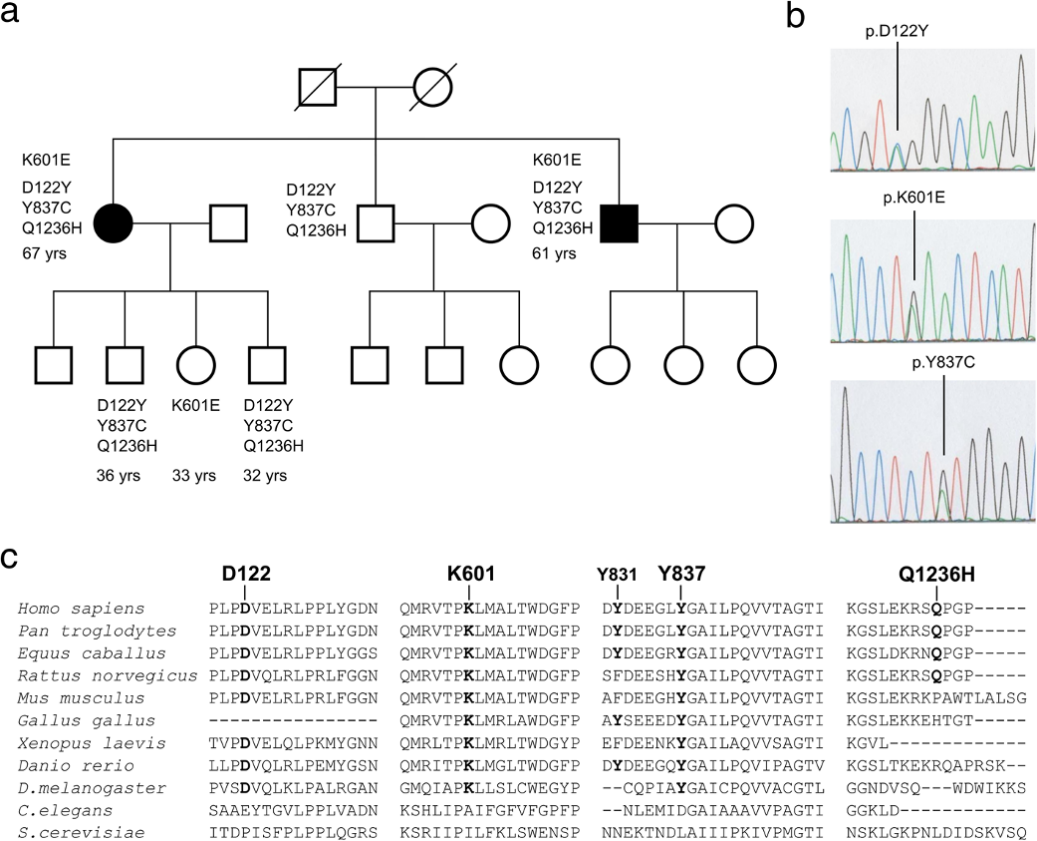
**Mutational screening of the** ***POLG*** **gene.** **a**, Pedigree of the family. Affected members with an adult onset mitochondrial ataxia syndrome are shown by solid symbols. The segregation pattern of the changes identified and the ages of the pedigree members under study are indicated. **b**, Identification of mutations in the *POLG* gene in two patients with mitochondrial ataxia syndrome. Electropherograms are shown indicating the nucleotide changes identified. **c**, Amino acid sequence alignments of PolGA that contain the residues D122, K601 and Y837 in which novel substitutions were identified, and the common polymorphism Q1236H are shown.

A 67 year-old woman (patient I-1, Figure 1) was referred to our hospital because of a progressive 15-year history of ataxia, dysarthria and sensory axonal polyneuropathy. There was no relevant history of neurological diseases in the patient’s family. At age 52, the patient developed instability upon walking and paraesthesia in lower limbs, when no other symptoms were apparent. Clinical examination at age 67 revealed dysarthria, gait ataxia, hyporeflexia and tactile hypoestesia. Blood tests excluded acquired ataxic syndromes. Molecular analyses excluded mutations in the *SCAS*, *frataxin* and *x*-*fragile* genes. Nerve conduction studies (NCS) showed absence of sural sensory action potentials. Cranial and spinal cord magnetic resonance imaging (MRI) displayed atrophy of the cerebellar hemispheres, high signals in the dentatum and thalamus nuclei, hyperintensities in images of the subcortical white substance, and supratentorial cortico-subcortical atrophy. Ten years later, dysarthria was more evident, and cognitive symptoms were noted. A neuropsychological examination showed a cortico-subcortical cognitive impairment, with defects in attention/concentration and memory tests. Subsequently, she developed a progressive left hemiparesis related to a non-traumatic subdural haematoma that required neurosurgical treatment, and began to experience myoclonus and tonic-clonic seizures. Antiepileptic treatment was started, resulting in complete seizure control. However, the ataxic gait and dysarthria worsened, and a weak bilateral palpebral ptosis was observed. A second NCS revealed a severe sensory axonal neuronopathy. Presently, the patient requires bilateral support on crutches.

The 61 year-old brother of the female patient showed a similar clinical presentation over a 7-year period (patient I-3, Figure 1). He began to present with an unsteady walk, and showed tremor in both legs when standing. In fact, he had to move to avoid instability with the tremors, suggesting an orthostatic tremor. Additionally, he stated that he had difficulty in concentrating. Clinical neurological examination revealed dysarthria, and no deep tendon reflexes were evident. Analytical blood tests were negative, and NCS revealed a sensory axonal neuropathy. Neuropsychological examination showed a normal cognitive function. MRI displayed high signals in the cerebellar hemispheres, thalami and spinal cord. Hyperintensities in images of the subcortical frontal and temporal white substances were also observed. Five years later, the patient began to show myoclonia and tonic-clonic seizures. EEG revealed an alpha rhythm intercalated with speak-wave paroxysms, predominantly in the frontal lobe. Light stimulation provoked the appearance of speak-wave discharges with systemic myoclonia, whereas sound stimulation did not evoke changes in the EEG. Antiepileptic treatment was initiated and seizures were controlled. At this time, the patient presents with a sensory axonal neuropathy in the lower limbs, moderate dysarthria, and an ataxic gait. The patient does not suffer any functional limitations and lives independently.

### Molecular genetic analysis

Samples from patients were obtained in accordance with the Helsinki Declaration of 1975, as revised in 2000. The Ethics Committees of the Puerta de Hierro Hospital approved the study.

Total DNA from patients and relatives was extracted from blood samples following written informed consent using a standard phenol-chloroform protocol. Subsequently, amplification by PCR of the coding exons and the flanking intronic sequences of the *POLG* gene was carried out. Following PCR amplification, direct sequencing of amplicons was performed on both strands in an ABI 3730 sequencer (Applied Biosystems; http://www.appliedbiosystems.com; Foster City, CA) using a dye terminator cycle sequencing kit (Applera, Rockville, MD). Primer sequences and PCR conditions are available upon request. For every sequence variant detected, a cohort of 200 ethnically matched control subjects was screened using the same methods. Multiple sequence alignment of PolGA sequences was performed using the Clustal W2 server (http://www.ebi.ac.uk/Tools/msa/clustalw2/).

### Computational analysis

To evaluate the likely pathogenicity of all the identified *POLG* gene sequence variants, a 3D structural analysis of the PolG protein was performed using the Alpers mutation clustering tool previously described by Euro et al. [12].

## Results and discussion

For genomic characterization, exons and flanking intronic regions of the *POLG* gene were PCR-amplified and sequenced in patients as described previously [10]. Both patients showed three novel heterozygous nucleotide changes in the *POLG* gene: c.364G > T (p.D122Y), c.1801A > G (p.K601E), and c.2510A > G (p.Y837C), as well as the known heterozygous polymorphism (c.3708G > T (p.Q1236H). As an indication of possible pathogenicity, these sequence variants were not found in 200 chromosomes from controls or in the available databases, and the three amino acids are evolutionarily conserved (Figure 1). The segregation pattern of these nucleotide variants in the family members for which we could obtain samples is shown in Figure 1.

The PolG enzyme has been studied extensively since its discovery, and the structure-function relationships among its domains are now well understood. PolG serves as the replicative DNA polymerase in mitochondria and its holoenzyme form comprises a catalytic subunit (PolGA) and a dimeric accessory subunit (PolGB), which enhances greatly the activity and processivity of the holoenzyme [13].

**Figure 2:**
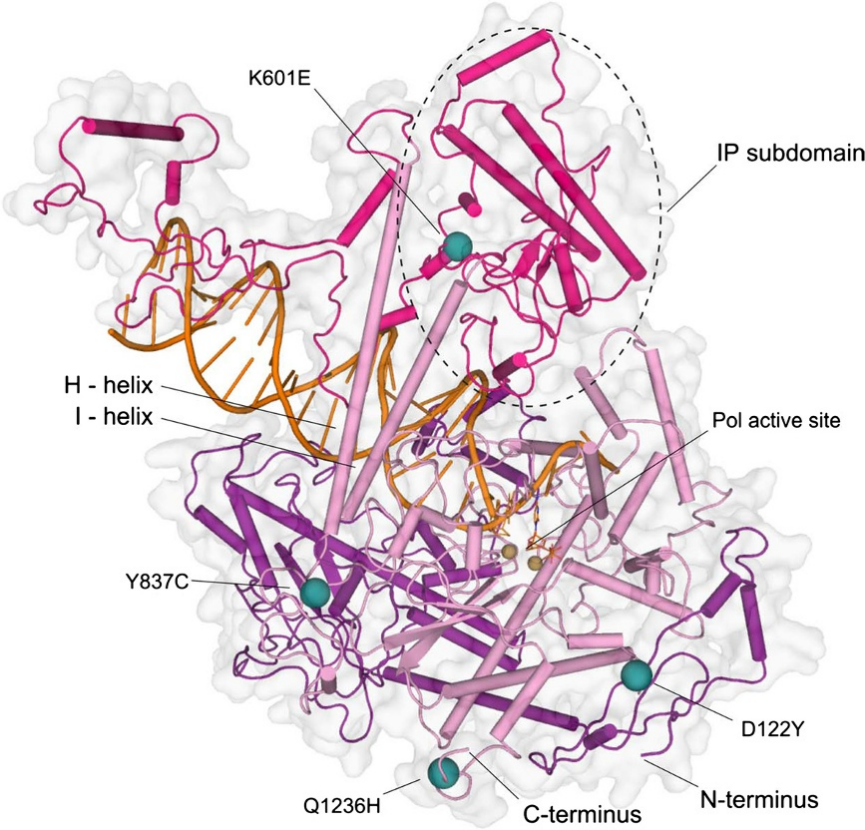
**Locations of the mutations in the PolG catalytic core with modeled primer template DNA.** The four mutations under study are distributed throughout the structure of PolG catalytic core. Residue K601 is buried within the spacer-region (magenta) IP subdomain. Residue Y837 is located within a region between the exonuclease (purple) and polymerase domains (pink), at a distance of ~29 Å from the polymerase active site The location of Y831 on the surface of the enzyme at the opposite side of the thumb helices (H, I) from the putative DNA-binding channel excludes it from any direct interactions with the polymerase active site. D122 resides in the N-terminal region of the exonuclease domain, but it is much closer structurally to the polymerase domain and the polymerase active site. Residue Q1236 resides within the C-terminus of the PolG enzyme. Primer-template DNA was modeled onto the crystal structure of PolG (PDB:3IKM) using the bacteriophage T7 DNA polymerase crystal structure (PDB:1T8E) as a template.

PolGA consists of three structural and functional domains [14]; Figure 2. The N-terminal exonuclease domain (exo) contains a 3’-5’ proofreading activity, which hydrolyzes nucleotides that are misincorporated during DNA synthesis, increasing several hundredfold the fidelity of mitochondrial DNA replication. The C- terminal polymerase domain (pol) contains the 5’-3’ DNA polymerase active site where incoming nucleotides are polymerized onto the nascent primer strand. The central spacer domain contains the major DNA binding interface of the holoenzyme, as well as the interaction region for binding the accessory subunit dimer and putatively, other protein partners of the mitochondrial DNA replisome.

Recent studies by Euro et al. and Farnum et al. evaluated the published reports of structure-function analyses of PolG by identifying ‘hot-spots” for pathogenic mutations within its three-dimensional structure [12, 15]. Critical functional regions of the PolGA structure were first reported as Alpers clusters, and thereafter demonstrated to represent a total of 136 pathogenic human mutations that give rise to the entire spectrum of POLG syndromes. Thus, the clustering model can be used as a guide for assessing the probability of the deleterious effects of mutations within the PolG structure.

**Figure 3:**
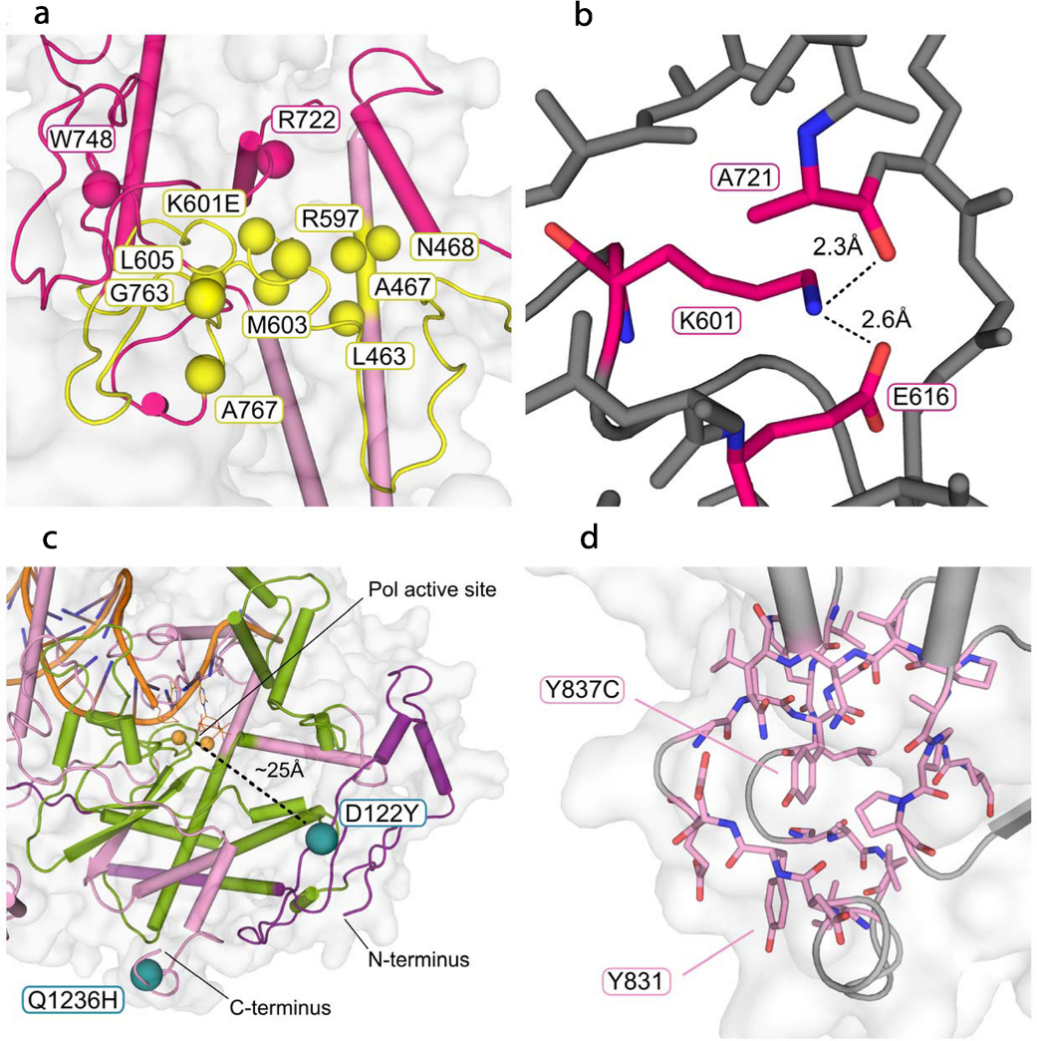
**Structural details of the four mutations under study.** **a**, ten residues in the spacer IP subdomain that map within 15 Å from K601 are known to have pathogenic human mutations. Yellow coloring shows pathogenic Alpers cluster 2 (DNA binding channel) as defined by Euro et al. [12]. The most common known *POLG* mutation, A467T, has been reported to have some dominant-like features [16]. **b**, K601 interacts with A721 (2.3 Å) and E616 (2.6 Å) in the crystal structure of PolGA (PDB:3IKM). The ionic interactions depicted by dashed lines cannot exist with the charge-reversal mutation K601E. **c**, mutation D122Y lies at the outer periphery of the pol active site access region, within ~25 Å from the active site. Green coloring shows Alpers cluster 1 residues. Cluster 1 contains all of the conserved Pol family A motifs, as well as all known pathogenic mutations in the fingers and palm subdomains of the pol domain. The C-terminus, where mutation Q1236H resides, is considerably further away from the regions that are likely to affect nucleotide polymerization. **d**, residue Y837 does not form any critical bonds in the PolGA structure. Its surrounding residues assert only limited spatial pressure, and replacement of this residue with a cysteine does not cause obvious effects on the tertiary structure. The nearest mutation studied previously, Y831C, is located six amino acids on the N-terminal side in the primary sequence.

Of the four mutations under study (Figure 2), our structural analysis suggests that the amino acid change K601E is the most likely to have deleterious effects. K601E is the only one of the mutations to have prominent binding partners in the crystal structure (A721 and E616; Figure 3b); these bonds cannot exist with the charge-reversal mutation from lysine to glutamate. As such, the c.1801A > G mutation is likely to cause significant structural changes within the intrinsic processivity (IP) subdomain of the spacer domain (Figure 2). Due to the central location of K601 within the spacer region, these changes are likely to affect both DNA binding and binding to the proximal accessory subunit, as well as to potentially hinder other putative protein-protein interactions within the mitochondrial replisome.

Residue K601 maps within pathogenic Alpers cluster 2 as defined by Euro *et al*. [12]. This cluster defines a high-risk area of the PolG tertiary structure for pathogenic mutations due to altered DNA binding affinity. This in turn leads to enzyme dysfunction, which results in various human mitochondrial pathologies ranging from early onset Alpers syndrome to late onset progressive external ophthalmoplegia, depending predominantly on the specific compound heterozygous mutations carried by the patient. In fact, ten known pathogenic amino acid substitutions within the spacer IP-subdomain map within 15 Å of residue K601 (Figure 3b; [15]). Of these ten amino acids, at least one (A467T) has been reported to present dominant-like inheritance patterns [16]. Dominant *POLG* mutations are rare among the known human pathogenic mutations, whereas pathogenicity is exhibited typically by recessive compound heterozygous mutations. The location of K601 within the PolG structure suggests that the observed phenotype may result from enzyme stalling during mitochondrial DNA replication. Enzyme stalling during replication has been proposed to result in a gradual mtDNA depletion in post-mitotic tissues, which can lead to a progressive, late-onset pathology [17]. This does not however, exclude the possibility that the additional remaining mutations under study contribute to the deleterious phenotype in our patients.

The c.3708G > T mutation causing the Q1236H amino acid substitution has been reported to be non-pathogenic in numerous studies [18], and its lack of conservation in evolution within the C-terminus of PolG (Figure 1c), which has no known functionality to date, suggests strongly that it does not contribute to a pathogenic phenotype. The dbSNP database reports its frequency in a control population of 225 individuals of northern and western European descent as high as 8.4% [19].

The Y837C mutation lies on the surface of the pol domain, in a region of the structure with no known critical functionality. This location excludes Y837 from any direct interactions with the residues at the polymerase active site. Furthermore, it does not have any prominent interacting partners within the PolGA crystal structure, and this region of the structure exhibits relaxed spatial restrictions (Figure 3d). Finally, a known, non-pathogenic mutation, Y831C, lies within the same region of the structure, at an alpha-carbon distance of 9.6 Å and only six amino acids away in primary sequence (Figure 1c). Y831C was reported originally as pathogenic [20], but later interpreted as non-deleterious [3, 21, 22]. Altogether these considerations argue against likely pathogenic effects of Y837C. At the same time however, the evolutionary conservation of Y837 gives some credence to a possible unknown role in enzyme folding or function, leaving its contribution to a pathogenic phenotype ambiguous.

Amino acid residue D122Y lies on the surface of the PolG enzyme within 25 Å from the pol active site (Figure 3c). Its location at the outer periphery of a region providing access to the pol active site for incoming nucleotides, makes it the second most likely candidate of the four mutations under study to give rise to deleterious effects. The relative proximity of D122Y to the pol active site, and to the region defined as Alpers cluster 1 (pol active site and its environs), suggests the possibility that it engenders defects in nucleotide polymerization. Cluster 1 contains all of the conserved family A polymerase motifs, as well as all the known pathogenic mutations in the palm and fingers subdomains of the pol domain of the enzyme.

Analysis of evolutionary sequence conservation shows that with the exception of nematodes, residue D122 is highly conserved in the eumetazoans and by comparison, residue Y837 is less well conserved (data not shown). Because D122 and Y837 are novel mutations that are not positioned within a known critical region for PolG functionality, nor do they have prominent interacting residue partners within the 3D structure, their possible conformational role in enzyme stability was estimated using PON-P [23]. PON-P is a sequence pathogenicity prediction engine that combines the results of multiple primary sequence pathogenicity prediction algorithms into a single consensus prediction. PON-P assigns residue D122Y a very low chance for pathogenicity (8%), and estimates its ability to predict Y837C pathogenicity as unreliable (data not shown). An additional element of uncertainty regarding D122Y are the 69 amino acid residues of the N-terminus that are missing from the PolG crystal structure (PDB:3IKM) [14]. These missing amino acids include the putative mitochondrial import sequence as well as a poly-Q stretch, in which variations have been associated with male infertility [24], though its role in PolG functionality remains elusive.

## Conclusions

In this study, three novel nucleotide changes and the known p.Q1236H polymorphism in the *POLG* gene have been identified in the two affected members of a family with a mitochondrial ataxia syndrome. A 3D structural analysis of the protein argues that the p.K601E mutation is likely to be the most significant contributing factor to a pathogenic phenotype, although the coexistence of all the identified *POLG* changes (allele1: p.K601E and allele 2: p. Y837C, p.D122Y, Q1236H) may be necessary to produce the late-onset mitochondrial ataxia phenotype observed.

### Consent

Written informed consent was obtained from the patient and each of the patient’s relatives who were involved in this study for publication of this article and any accompanying images. A copy of the written consent is available for review by the Editor-in-Chief of this journal.

## Abbreviations

POLG: DNA polymerase gamma
MIRAS: Mitochondrial recessive ataxia syndrome symptoms
mtDNA: Mitochondrial DNA
NCS: Nerve conduction studies
MRI: Magnetic resonance imaging
EEG: Electroencephalogram.

## References

[1] Naviaux RK, Nguyen KV (2005). POLG mutations associated with Alpers syndrome and mitochondrial DNA depletion. Ann Neurol.

[2] Spinazzola A, Zeviani M (2009). Disorders from perturbations of nuclear-mitochondrial intergenomic cross-talk. J Intern Med.

[3] Wong LJ, Naviaux RK, Brunetti-Pierri N, Zhang Q, Schmitt ES, Truong C, Milone M, Cohen BH, Wical B, Ganesh J, Basinger AA, Burton BK, Swoboda K, Gilbert DL, Vanderver A, Saneto RP, Maranda B, Arnold G, Abdenur JE, Waters PJ, Copeland WC (2008). Molecular and clinical genetics of mitochondrial diseases due to POLG mutations. Hum Mutat.

[4] Copeland WC (2012). Defects in mitochondrial DNA replication and human disease. Crit Rev Biochem Mol Biol.

[5] Suomalainen A, Isohanni P (2010). Mitochondrial DNA depletion syndromes–many genes, common mechanisms. Neuromuscul Disord.

[6] Van Goethem G, Dermaut B, Lofgren A, Martin JJ, Van Broeckhoven C (2001). Mutation of POLG is associated with progressive external ophthalmoplegia characterized by mtDNA deletions. Nat Genet.

[7] Hakonen AH, Heiskanen S, Juvonen V, Lappalainen I, Luoma PT, Rantamaki M, Goethem GV, Lofgren A, Hackman P, Paetau A, Kaakkola S, Majamaa K, Varilo T, Udd B, Kaariainen H, Bindoff LA, Suomalainen A (2005). Mitochondrial DNA polymerase W748S mutation: a common cause of autosomal recessive ataxia with ancient European origin. Am J Hum Genet.

[8] Hakonen AH, Goffart S, Marjavaara S, Paetau A, Cooper H, Mattila K, Lampinen M, Sajantila A, Lonnqvist T, Spelbrink JN, Suomalainen A (2008). Infantile-onset spinocerebellar ataxia and mitochondrial recessive ataxia syndrome are associated with neuronal complex I defect and mtDNA depletion. Hum Mol Genet.

[9] Winterthun S, Ferrari G, He L, Taylor RW, Zeviani M, Turnbull DM, Engelsen BA, Moen G, Bindoff LA (2005). Autosomal recessive mitochondrial ataxic syndrome due to mitochondrial polymerase gamma mutations. Neurology.

[10] Posada IJ, Gallardo ME, Dominguez C, Rivera H, Cabello A, Arenas J, Martin MA, Garesse R, Bornstein B (2010). [Mitochondrial DNA depletion and POLG mutations in a patient with sensory ataxia, dysarthria and ophthalmoplegia]. Med Clin (Barc).

[11] Saneto RP, Cohen BH, Copeland WC, Naviaux RK (2013). Alpers-Huttenlocher syndrome. Pediatr Neurol.

[12] Euro L, Farnum GA, Palin E, Suomalainen A, Kaguni LS (2011). Clustering of Alpers disease mutations and catalytic defects in biochemical variants reveal new features of molecular mechanism of the human mitochondrial replicase, Pol gamma. Nucleic Acids Res.

[13] Kaguni LS (2004). DNA polymerase gamma, the mitochondrial replicase. Annu Rev Biochem.

[14] Lee YS, Kennedy WD, Yin YW (2009). Structural insight into processive human mitochondrial DNA synthesis and disease-related polymerase mutations. Cell.

[15] Farnum GA, Nurminen A, Kaguni LS (1837). Mapping 136 pathogenic mutations into functional modules in human DNA polymerase gamma establishes predictive genotype-phenotype correlations for the complete spectrum of POLG syndromes. Biochim Biophys Acta.

[16] Luoma PT, Luo N, Loscher WN, Farr CL, Horvath R, Wanschitz J, Kiechl S, Kaguni LS, Suomalainen A (2005). Functional defects due to spacer-region mutations of human mitochondrial DNA polymerase in a family with an ataxia-myopathy syndrome. Hum Mol Genet.

[17] Ashley N, O’Rourke A, Smith C, Adams S, Gowda V, Zeviani M, Brown GK, Fratter C, Poulton J (2008). Depletion of mitochondrial DNA in fibroblast cultures from patients with POLG1 mutations is a consequence of catalytic mutations. Hum Mol Genet.

[18] Di Fonzo A, Bordoni A, Crimi M, Sara G, Del Bo R, Bresolin N, Comi GP (2003). POLG mutations in sporadic mitochondrial disorders with multiple mtDNA deletions. Hum Mutat.

[19] Wheeler DL, Barrett T, Benson DA, Bryant SH, Canese K, Chetvernin V, Church DM, DiCuccio M, Edgar R, Federhen S, Geer LY, Kapustin Y, Khovayko O, Landsman D, Lipman DJ, Madden TL, Maglott DR, Ostell J, Miller V, Pruitt KD, Schuler GD, Sequeira E, Sherry ST, Sirotkin K, Souvorov A, Starchenko G, Tatusov RL, Tatusova TA, Wagner L, Yaschenko E (2007). Database resources of the National Center for Biotechnology Information. Nucleic Acids Res.

[20] Mancuso M, Filosto M, Oh SJ, DiMauro S (2004). A novel polymerase gamma mutation in a family with ophthalmoplegia, neuropathy, and Parkinsonism. Arch Neurol.

[21] Luoma PT, Eerola J, Ahola S, Hakonen AH, Hellstrom O, Kivisto KT, Tienari PJ, Suomalainen A (2007). Mitochondrial DNA polymerase gamma variants in idiopathic sporadic Parkinson disease. Neurology.

[22] Woodbridge P, Liang C, Davis RL, Vandebona H, Sue CM (2013). POLG mutations in Australian patients with mitochondrial disease. Intern Med J.

[23] Olatubosun A, Valiaho J, Harkonen J, Thusberg J, Vihinen M (2012). PON-P: integrated predictor for pathogenicity of missense variants. Hum Mutat.

[24] Rovio AT, Marchington DR, Donat S, Schuppe HC, Abel J, Fritsche E, Elliott DJ, Laippala P, Ahola AL, McNay D, Harrison RF, Hughes B, Barrett T, Bailey DM, Mehmet D, Jequier AM, Hargreave TB, Kao SH, Cummins JM, Barton DE, Cooke HJ, Wei YH, Wichmann L, Poulton J, Jacobs HT (2001). Mutations at the mitochondrial DNA polymerase (POLG) locus associated with male infertility. Nat Genet.

